# A Review of Natural Fibres and Biopolymer Composites: Progress, Limitations, and Enhancement Strategies

**DOI:** 10.3390/ma17194878

**Published:** 2024-10-04

**Authors:** Innes McKay, Johnattan Vargas, Liu Yang, Reda M. Felfel

**Affiliations:** Advanced Composites Group, Mechanical and Aerospace Engineering, University of Strathclyde, Glasgow G1 1XJ, UK; j.vargas@strath.ac.uk (J.V.); l.yang@strath.ac.uk (L.Y.)

**Keywords:** natural fibres, flax fibre, biopolymers, bio-composites, surface treatments

## Abstract

The interest in natural fibres and biopolymers for developing bio-composites has greatly increased in recent years, motivated by the need to reduce the environmental impact of traditional synthetic, fossil fuel-derived materials. However, several limitations associated with the use of natural fibres and polymers should be addressed if they are to be seriously considered mainstream fibre reinforcements. These include poor compatibility of natural fibres with polymer matrices, variability, high moisture absorption, and flammability. Various surface treatments have been studied to tackle these drawbacks, such as alkalisation, silane treatment, acetylation, plasma treatment, and polydopamine coating. This review paper considers the classification, properties, and limitations of natural fibres and biopolymers in the context of bio-composite materials. An overview of recent advancements and enhancement strategies to overcome such limitations will also be discussed, with a focus on mechanical performance, moisture absorption behaviour, and flammability of composites. The limitations of natural fibres, biopolymers, and their bio-composites should be carefully addressed to enable the widespread use of bio-composites in various applications, including electronics, automotive, and construction.

## 1. Introduction

In recent years, the composites sector has recognised the need for a new generation of renewable composite materials, and research into the use of sustainably derived polymers and fibre reinforcements has dramatically increased. Glass fibre is the most widely used reinforcement in polymer composites [1]; however, their production requires high amounts of energy, and they make disposing of composite components a challenge [2]. The aim of utilising natural fibres and biopolymer matrices is to reduce the environmental impact associated with composite material production, use, and disposal [3].

Flax fibre is one of the most heavily researched natural fibres due to its widespread availability, temperate growing conditions, and high reported specific mechanical properties [4]. However, there are several key shortcomings associated with flax fibre that currently prevent its widespread use as a reinforcing material. Such limitations include variability in properties due to the variety of growing conditions and processing [5], poor interfacial strength with polymer matrices [6], high moisture absorption [7], and thermal stability [8]. These limitations should be suitably addressed if flax fibre is to be considered a mainstream fibre reinforcement.

The chemical and physical treatments of plant fibres offer a potential solution for enhancing bio-composite performance while reducing moisture absorption and flammability [9]. Although the resulting properties of treated composites are promising, such improvements are still marginal [10]. Hence, the identification and characterisation of novel treatment processes/chemicals are required to further address the limitations of natural fibres and improve the performance and versatility of their bio-composites.

This paper provides an overview of natural fibres, biopolymers, and their bio-composites, with a focus on current limitations and enhancement approaches. The first section presents background on natural fibres, discussing their classification and source before focusing on the properties, limitations, and enhancement techniques. Section two outlines the synthesis routes of various noteworthy biopolymers and considers the challenges related to the use of biopolymers in applications such as packaging and automotive, highlighting potential solutions. Finally, section three examines the properties, current limitations, and enhancement strategies of bio-composites, discussing their mechanical properties, thermal stability, moisture absorption behaviour, and flammability.

## 2. Natural Fibres

### 2.1. Classification of Natural Fibres

**Figure 1:**
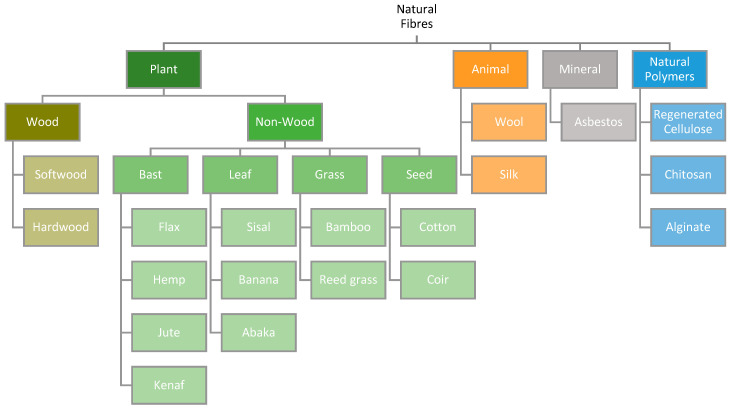
Classification of common natural fibres by origin.

Natural fibres are classified based on the source from which they are derived, namely plants, animals, or minerals. Some natural fibres that have been considered for reinforcement in bio-composites are shown in Figure 1.

#### 2.1.1. Plant-Based Fibres

**Figure 2:**
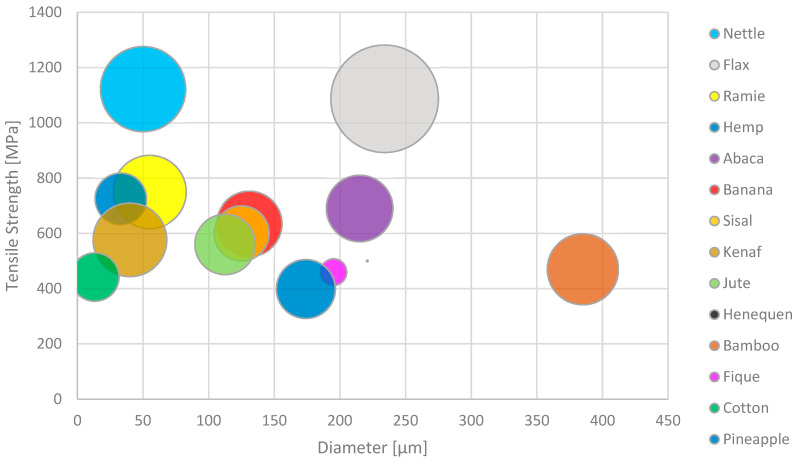
Tensile strength vs. diameter of common plant fibres.

Plant fibres that are not derived from wood may be further sub-categorised by the part of the plant from which they are extracted, namely bast stem, leaf, grass, and seeds. The typical tensile properties of various plant fibres are summarised in Table 1 and Figure 2.

##### Flax

**Figure 3:**
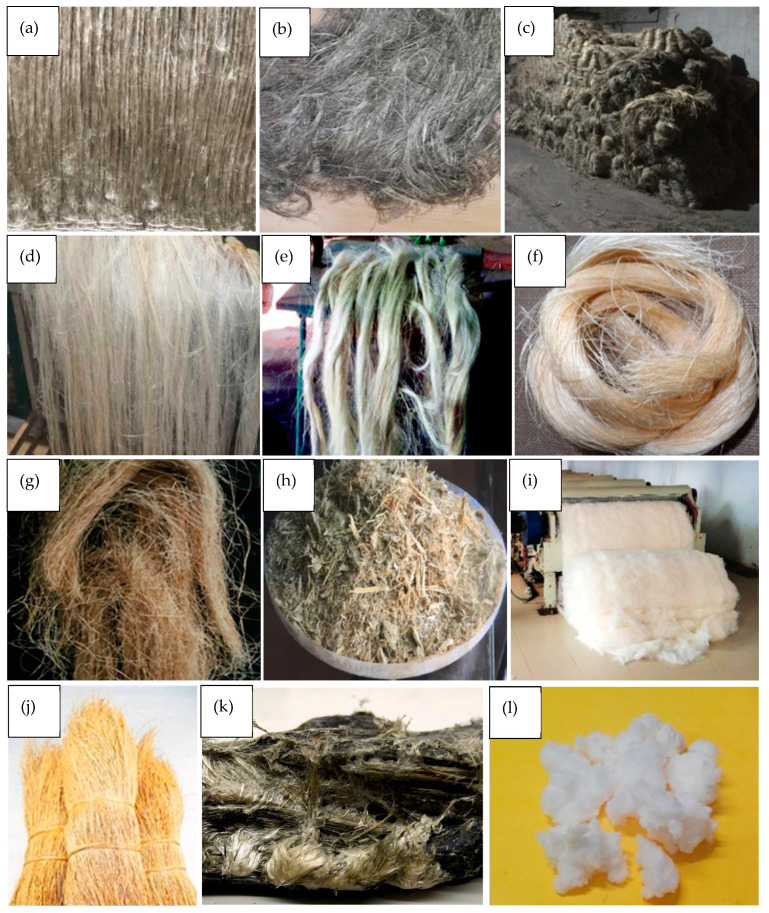
Extracted fibres of flax (**a**) [40], hemp (**b**) [41], jute (**c**) [42], kenaf (**d**) [42], sisal (**e**) [42], abaca (**f**) [42], bamboo (**g**) [43], bagasse (**h**) [42], cotton (**i**) [42], coir (**j**) [32], asbestos (**k**) [44], and regenerated cellulose fibres (**l**) [45].

Varieties of flax (linum ustatissimum) are grown across the world to produce long fibres for textiles and linseed oil for food and chemical applications (Figure 3). Europe is the largest global producer of long flax fibre, with production largely concentrated in France, Belgium, and the Netherlands. Once harvested, flax straw is retted to separate the fibres from the woody core and then scutched and hackled to extract long, fine fibres for yarn. Flax is widely used in the textile industry to make linen fabrics, as well as woven cloth, yarn, and rope. It has also recently been used as a reinforcement in bio-composites for the automotive, aeronautical, and construction industries [22].

##### Hemp

Hemp is a bast fibre with a similar structure and properties as flax; however, it is not produced on such as large scale due to strict government regulations. China is the world’s largest producer of hemp, with 65,000 ha of hemp cultivated in 2021, only 18.5% of which was for use in textile applications [23]. Like flax, hemp fibre has seen an increased interest in recent years as a sustainable reinforcement in polymer composites; however, it has also found applications in insulation, paper, and textile sectors [24].

##### Jute

Jute is the most widely produced bast fibre and the second most produced natural fibre worldwide following cotton. The majority of jute is produced in South Asia, in countries such as Bangladesh, India, and Nepal, with the region’s warm and humid climate providing optimal conditions for cultivation. Conventional applications for jute fibre include woven bags for transporting agricultural and industrial goods and as a reinforcement material to strengthen sub-surface soil during the construction of roads. Jute has also been considered as a potential sustainable fibre reinforcement alternative to glass fibre in automotive applications [25].

##### Kenaf

Kenaf is a fast-growing, low-cost crop grown primarily in India and China. Kenaf is grown for both its seeds and fibres, widely utilised in food production as well as paper and textile industries. The versatility and resilience of Kenaf compared to other bast fibre crops make it an attractive natural fibre for use in composite materials [26].

##### Sisal

Sisal is a large shrub with thick, spirally arranged leaves belonging to the agave family. Although native to Mexico, sisal is mostly cultivated in semi-arid regions of Brazil, East Africa, and China. Sisal only requires 400 mm of rainfall a year, making it resilient to drought; however, its growth rates are extremely slow compared to the bast crops. Sisal fibre is commonly used in textile applications; however, it is also viewed as a potential reinforcing material in bio-composites [27].

##### Abaca

Abaca is a leafy tropical plant that is produced in the Philippines and certain regions of Central America. The extraction of abaca fibres is a three-stage process that initially involves the separation of inner and outer leaves, stripping of unwanted pulpy material, and finally, drying of the extracted fibres. Currently, the primary application of abaca fibre is as a construction material, where it is used in the production of composite wall panels, roofing, and flooring [28].

##### Bamboo

Most of the global supply of bamboo is produced in Asia, largely in China; however, it is also produced in parts of South America and Africa. Bamboo is very diverse and versatile, being able to sustain fast growth in a variety of climatic conditions. The total cultivated area of ‘Moso’ bamboo, the species most commonly used for the production of bamboo fibre, covers approximately 3 million hectares worldwide. The fibre extraction process involves splitting whole stalks into thin slivers, which are then soaked in a solution approved by the Global Organic Textile Standard to allow the easy separation of fibres. Current common applications of bamboo fibres include household items and textile fabrics to produce clothing; however, they are also widely used as a source of cellulose from which regenerated rayon fibres are produced [29].

##### Bagasse

Bagasse is an agricultural by-product generated through the extraction of juice from sugarcane stalks, grown in regions with warm, humid climates such as southeast Asia, Brazil, and India. Recently, bagasse fibres have been identified as a potential reinforcing material in construction applications to replace hardwood in composite structural and insulation panels [30].

##### Cotton

Cotton is the most widely produced natural fibre in the world, with 26 million tonnes of textile fibres produced in 2018. Once harvested, the cotton is dried and separated from seeds and other unwanted plant materials. Cotton is widely used in the textile industry for the manufacture of clothes and other products [31].

##### Coir

Coconut coir is a short, fibrous substance that surrounds the hard internal shell of a coconut seed. Coir fibre is produced in tropical/sub-tropical countries such as India, Sri Lanka, and Vietnam as a by-product of coconut harvesting. Once harvested, the coir is retted in water for 4–12 months, following which it is dried and removed from the outer husk. Fine white fibres, as shown in Figure 3, are often used for household items such as brushes and yarn, whereas stronger coarse brown fibres can be used as insulation or as a construction material [32].

#### 2.1.2. Animal Fibres

Animal fibres are composed of proteins such as keratin or collagen that are naturally produced by mammals and insects. The most recognisable animal fibre is wool, a keratin-based fibre that has been harvested from the coats of sheep for centuries to produce textiles [33].

##### Wool

Wool is produced globally in over 100 different countries, with Australia, China, and New Zealand leading the world in annual production [33]. Commercial applications for wool are largely associated with the production of clothing and other textiles; however, there has been recent interest in the utilisation of coarser waste wool as an acoustic and thermal insulator. Additionally, its use in hybrid composites has been investigated to utilise its inherent fire-retardant properties [34].

##### Silk

Silk is produced by a variety of insects, such as spiders and worms, to construct webs and cocoons. The most common form of silk is harvested from silkworms and has long been used in the textile industry to produce fine, durable garments. Like Keratin, silk is a fibrous protein biopolymer with relatively high failure strain and low stiffness compared to fibres commonly used as composite reinforcements [35]. The price of silk currently limits its applications to mostly high-end textiles; however, evaluation into the use of waste fabric has also been carried out [36].

#### 2.1.3. Mineral Fibres

The term ‘mineral fibres’ refers to a wide variety of materials, ranging from specific minerals that possess a ‘needle-like’ crystalline form (e.g., asbestos) to synthetic fibres extruded from molten material (e.g., basalt).

##### Asbestos

Asbestos is a generic term used to describe a variety of mineral species whose microstructure is composed of thin crystalline fibrils, as shown in Figure 3. Asbestos fibres were utilised widely in the construction industry during the 20th century for their high strength, flexibility, and heat resistance. However, asbestos is currently banned in almost 30% of the world due to serious health concerns related to its inhalation [37].

#### 2.1.4. Natural Polymer Fibres

Natural polymers extracted from bio-resources can be subsequently regenerated and drawn into fibres via wet spinning methods. Such methods have been employed for several decades to manufacture rayon fibres; a form of regenerated cellulose used within the fashion industry.

##### Regenerated Cellulose

Regenerated cellulose fibres (Figure 3) have recently garnered attention as a sustainable alternative to synthetic polymer fibres for textile applications. Multiple processes have been developed for spinning fibres with a variety of properties, with current research focused on the use of environmentally friendly dissolution agents. For example, Moriam et al. [38] produced Ioncell fibres from Kraft cellulose pulp with a tensile strength of up to 925 MPa.

##### Alginate

Alginate is a natural polysaccharide polymer commonly extracted from brown algae seaweed. Sodium alginate can be dissolved in water and subsequently coagulated in a metal ion solution such as calcium chloride to obtain insoluble fibres. It has been extensively used in the medical and food industries as a sorption medium in wound dressings and as a stabiliser, respectively [39].

##### Chitosan

Chitosan is obtained by the deacetylation of chitin, an abundant biopolymer extracted from the shells of molluscs and crustaceans. As a polysaccharide, chitosan possesses similar physical and chemical characteristics to cellulose, and its inherent antibacterial properties have led to its application in the bio-medical industry for wound care and drug delivery [39].

Flax fibre presents as the most promising natural fibre owing to its high specific mechanical properties, temperate growing climate, low cost, and sustainable production [46]. The following section gives a brief background on the production, structure, and properties of flax fibre and highlights several limitations that currently limit the use of plant fibres such as flax as reinforcements in composite materials.

### 2.2. Flax Fibre

The properties of flax fibres largely depend upon the complex internal structure of their plant cells [47]. Development of the flax plant cell is influenced by many agricultural and environmental factors such as seed variety, sowing period, fertiliser requirements, crop maturity, extraction method, temperature, humidity, and rainfall [48]. A clear understanding of the impact these factors have on the properties of flax fibres is thus essential in achieving consistent behaviour.

#### 2.2.1. Cultivation and Harvesting

The mechanical properties of flax fibre are influenced by many agricultural and environmental factors such as seed variety, sowing period, fertiliser requirements, crop maturity, and cultivation conditions. Optimum temperatures between 18.0 and 20.0 °C and annual precipitation of 600–650 mm have been reported [49]; however, these conditions vary according to region and variety. Additional factors such as soil composition and nutrient availability also impact the properties and yield of flax fibres; as such, fertilisers and pesticides are commonly utilised during cultivation.

#### 2.2.2. Processing

Immediately following harvest, flax straw is then baled in preparation for retting, a process employed to separate flax fibres from the woody stem of the plant. During the retting process, adhesive compounds such as pectin and hemicellulose decompose via microbial or enzymatic action, allowing for the easy separation of flax fibres from associated co-products.

Dew retting is the most widely practised retting technique in Europe and involves laying bundles of fibre in a recently harvested field for approximately two months. The process relies upon the natural growth of bacteria and fungus upon the flax surface in the presence of dew or rain to decompose the pectin bindings between fibre bundles. This process is considered the most environmentally friendly retting technique; however, its overall effectiveness is highly dependent on climatic conditions. Other methods, such as hot water or enzymatic retting, offer reduced processing times and higher fibre quality. However, these methods also require more energy, generate aqueous waste, and incur additional costs [50].

Dry retted flax is then drawn through toothed rollers to remove short fibres and unwanted stems in a process known as scutching. Scutched flax fibres are then hackled (i.e., combed) to remove any remaining residues, leaving long ‘slivers’ of flax fibre. Flax slivers can then be spun into continuous yarns and drawn into a highly aligned roving to produce woven fabrics or textiles [50].

Extracted flax fibres can be further processed via chemical extraction methods to obtain fibrous cellulose. This method has recently been employed to produce non-woven mats made from a mixture of cellulose and viscose fibres [51].

#### 2.2.3. Structure

**Figure 4:**
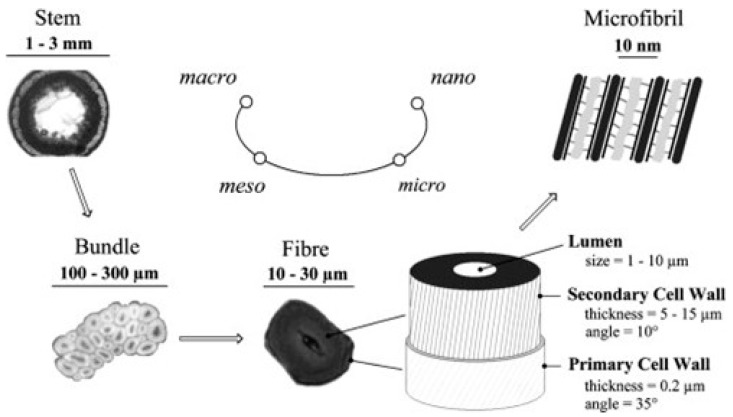
Hierarchical structure of flax fibre, from bast stems to elementary fibres [5].

Elementary flax fibres are 1.00–3.00 cm long plant cells with diameters of 10.0 – 30.0 µm, as shown in Figure 4. The plant cell wall has a hierarchical structure, with each concentric layer resembling a composite-like structure comprised of crystalline cellulose microfibrils embedded in an amorphous hemicellulose/lignin matrix. The thin outer (primary) wall contains randomly oriented microfibrils, whereas the cellulose structure in the secondary cell wall is more aligned. The S2 (secondary) sub-layer accounts for 80% of the total cell wall thickness and largely determines the mechanical properties of the elementary fibre. It is composed of highly aligned crystalline cellulose oriented along the longitudinal fibre axis, helically wound at an angle of between 5.00 and 10.0°. Elementary flax fibres are between 30.0 and 40.0 mm long and are arranged together in bundles between 10 and 40, bound together by a thin pectin layer to form “technical” fibres. Technical flax fibres can range from 60.0 to 140 cm in length, depending on the height of the plant stems at the time of harvest, and have a diameter between 100 and 300 µm [5].

#### 2.2.4. Properties of Flax Fibres

##### Mechanical Properties

**Figure 5:**
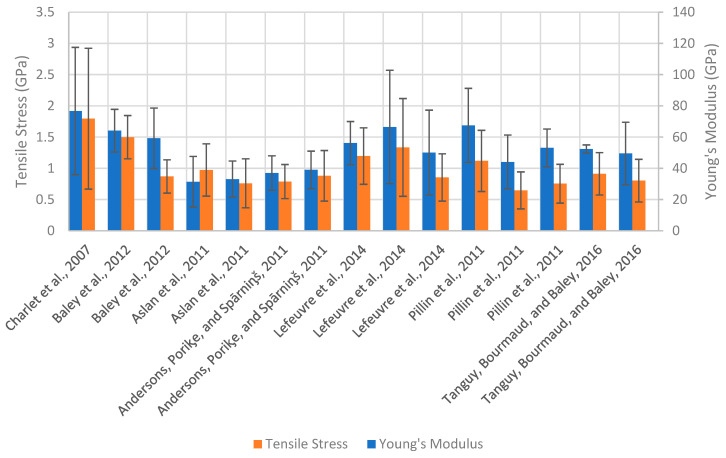
Average tensile properties of flax fibre from the literature [5,47,52,53,54,55,56].

A summary of the tensile properties of various elementary flax fibres as reported in the literature [5,47,52,53,54,55,56] is shown in Figure 5 and Table 2. Mechanical fibre extraction methods refer to traditional scutching and hackling processes, while cottonisation refers to fibres subjected to additional mechanical separation processes to produce fine, soft fibres (e.g., cutting, carding, and combing) [57]. Variations in both physical and mechanical properties can be seen across all varieties of flax due to differences in cultivation conditions between location and year. Additional factors such as differences in fibre extraction method, stem location, and defects obtained during decortication can also explain the large variations seen in the strength and modulus of flax fibre.

##### Thermal Properties

A summary of the thermal degradation properties of flax fibre obtained via thermogravimetric analysis is shown in Table 3. Initial weight loss occurring below 150 °C can be attributed to the evaporation of moisture present in the fibre. Subsequent degradation stages are associated with the decomposition and depolymerisation of specific plant cell constituents: pectin and hemicellulose between 200 and 260 °C, cellulose between 240 and 350 °C, and lignin in the range of 280–500 °C [58]. Retting technique and chemical treatments such as alkalisation can be seen to affect the temperature at which the maximum rate of degradation occurs (T_max_), and the weight loss associated with each stage of degradation (Table 3) as they remove the less thermally stable hemicellulose, pectin, and waxes [59].

### 2.3. Limitations

#### 2.3.1. Variability

One of the notable limitations of flax fibre is the variability of its mechanical properties. Cultivation conditions and processing methods all have an impact on the final properties of flax fibre, making it difficult to achieve consistency between fibres extracted from different harvests.

##### Cultivation Conditions

The climatic conditions in which the flax is cultivated and grown have been shown to have a significant effect on the final strength of extracted fibres. Pisupati et al. [48] examined the effect of location and climate conditions on the yield and fibre strength of ten different varieties of flax grown in France across two years. It was observed that in 2017, unfavourable temperatures and precipitation at each location during the early stages of growth resulted in a poorer yield of long fibres and decreased average bundle strength. Such differences were also noted in 2018, with flax fibre from the location that received optimum growth conditions exhibiting superior bundle strength. The differences in fibre properties were attributed to an underdeveloped cell wall structure directly associated with unfavourable climatic conditions during the early stages of growth.

It is known that the available nutrient content within the soil can greatly affect the final properties of extracted flax fibres [63]. The optimal soil fertility is dependent on regional climate conditions and so nutrient requirements vary with both location and planting history. Unfavourable temperature and precipitation levels can affect the dynamics of nutrients within the soil, leading to fluctuations in soil fertility and fibre yield [64].

##### Processing Methods

Retting duration is known to play a significant role in the mechanical properties of the resultant flax fibre; however, the unpredictability in environmental conditions makes optimisation difficult. Thus, although subject to greater variations due to changes in environmental conditions, the extended processing times associated with dew retting allow for slightly more control of retting duration, reducing the likelihood of fibre degradation due to over-retting [65]. Similarly, the effectiveness of water retting is largely dependent on the environmental conditions and eco-system of the given body of water, with temperature, vegetation, dissolved oxygen, and microbial activity all affecting the final properties of the extracted fibres. The use of storage tanks results in greater control of the process temperature, composition of water, and retting duration, allowing for process optimisation and a higher-performing flax fibre at additional cost and complexity [66].

##### Stem Location

Due to the nature in which flax fibre is produced, its mechanical properties can also vary along its length. Charlet et al. [5] compared the tensile properties of Hermès flax fibre obtained from three distinct stem sections: top, middle, and bottom. The mean diameter of the flax fibre was found to vary with its location in the stem, increasing from the bottom section to the top. The diameter was found to vary significantly within a few millimetres. The average tensile strength of fibres from the top, middle, and bottom portions of the stem was found to be 1340 (±470), 1800 (±1130), and 757 (±249) MPa, respectively. A similar trend was also observed for Young’s modulus. This was attributed to the impact of unfavourable growth conditions during the early stages of cultivation on the development of the secondary cell wall in older stem sections.

Goudenhooft et al. [67] investigated the change in properties of flax fibres during the early (60 days) and mature (120 days) development stages. For a 60-day-old flax plant, progressive stages of cell wall thickening are visible throughout the stem, from top to bottom. Top stem cell walls are characterised by loosely packed cellulose microfibril separated by pectic galactan chains. Mid-stem sections show a transitional thickening stage, whereby a partial packing of cellulose microfibrils in the outer secondary cell wall is observable. Bottom-stem sections show a fully mature and homogenised structure of compacted cellulose microfibrils. Each development stage is characterised by an increase in the indentation modulus in the cell wall, from 13.0 ± 2.50 GPa at the top of the stem to 19.5 ± 2.50 GPa at the bottom. Once fully mature, the indentation modulus of the top, middle, and bottom stem sections were found to be homogenous; however, variation between plants still exists. Similarly, the tensile strength of mature fibres was found to be much higher than developing ones, with values of 680 ± 337 MPa and 965 ± 302 MPa obtained, respectively.

#### 2.3.2. Defects

The decortication process, from retted straw to long flax fibres, has been shown to result in the formation of defects in the fibre structure known as kink bands [68] due to excessive bending and compressive loads. Large buckling failures in the secondary cell wall that cross the entire fibre diameter have been shown to cause protrusions in the primary cell wall, recognisable as a thickened band wrapping around the circumference of an elementary fibre, commonly known as a kink band. The introduction of defects in the secondary cell wall weakens the fibre within this region, creating a stress concentration at which fracture is likely to occur [69]. Manual decortication processes have been shown to reduce the occurrence of kink bands, resulting in higher values of tensile strength and elongation at break; however, this is not a practical method to implement at an industrial scale [70].

### 2.4. Summary

In summary, natural fibres can be classified based on the source from which they are extracted: plants, animals, or minerals. Plant fibres can be further categorised based on the type of vegetation from which they originate, e.g., bast, leaf, grass, or seed fibres. Flax fibre presents as the most promising bio-based alternative to E-glass due to its high specific tensile properties and low cost. Elementary flax fibres are composed of cellulose microfibrils embedded in an amorphous hemicellulose/lignin matrix, resembling a fibre-reinforced composite structure. Within plant stems, elementary flax fibres are arranged together in overlapping bundles of 10–40, bound together by a thin pectin layer to form technical fibres. These technical fibres are extracted from harvested flax via dew or water retting techniques and further processed into yarns and woven mats. Differences in variety, cultivation conditions, and processing methods can all affect the final tensile properties of flax fibre, making them highly subject to variation.

## 3. Biopolymers

**Figure 6:**
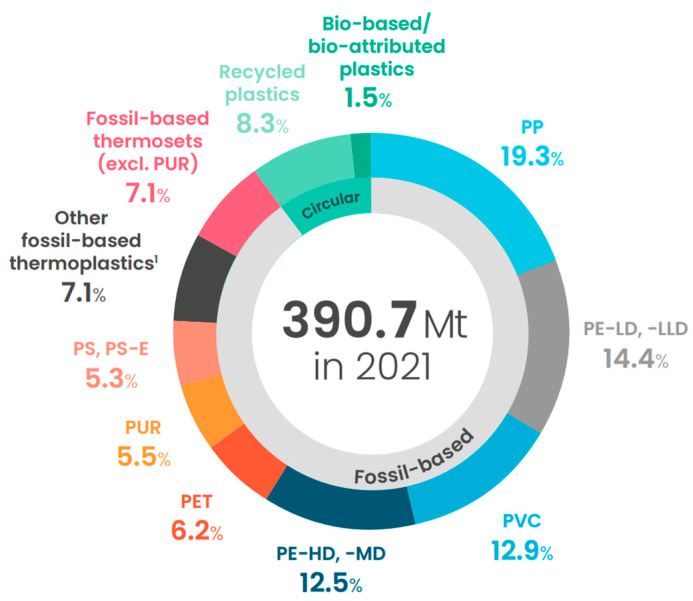
Global plastic production in 2021 by polymer type and origin [71].

As shown in Figure 6, fossil fuel-derived polymers currently account for approximately 90% of global plastic production, with only 1.5% being derived from bio-based resources [71]. Research into biopolymer replacements for commodity polymers has therefore garnered significant interest in recent years ahead of future plans to decrease global reliance on fossil fuels and reduce plastic pollution.

### 3.1. Definition of Biopolymer

**Figure 7:**
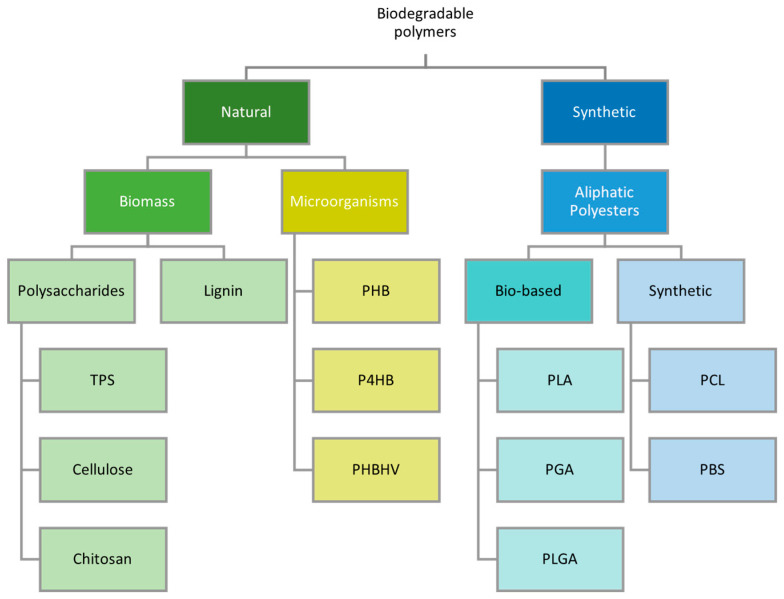
Classification of common biodegradable polymers by origin.

At least one of two criteria must be satisfied for a polymeric material to be classified as a “biopolymer”: it must be derived from bio-based natural resources or be biodegradable. As such, a biopolymer may fall into three distinct categories: natural and biodegradable, synthetic and biodegradable, and natural and non-biodegradable [72]. A summary of the classification of various biodegradable polymers based on origin is shown in Figure 7.

### 3.2. Biodegradability and Compostability

Both biodegradable and compostable materials degrade via microbial attack into natural substances such as water, carbon dioxide, methane, and residual biomass. The duration and conditions under which different biodegradable materials break down vary; however, it is generally agreed that this must take place at such a rate so as to avoid accumulation within the given waste stream [73]. In the case of composting, degradation proceeds under controlled conditions (i.e., in an industrial composting facility) at a rate specified within ASTM D6400 [74] and ISO 17088 standards [75]. The residual biomass generated as a result of the degradation process must also be benign with regard to its effect on the bioactivity and chemical quality of the resultant composting media [76].

There are a variety of biodegradation mechanisms that occur simultaneously in nature, depending on the environmental conditions. Biotic degradation mechanisms are driven by microorganisms such as bacteria or fungi secreting enzymes and other chemicals that degrade the polymer. Abiotic biodegradation proceeds via simple hydrolysis, photolysis, and thermal effects. These reactions can occur throughout the bulk material, whereas microbiological attack progresses inward from the surface [76]. Whether or not a polymer will degrade in a certain environment is determined by its physical and chemical characteristics: morphology, melting temperature, crystallinity, chemical functionality, and molecular weight [77].

### 3.3. Synthetic Biopolymers

#### 3.3.1. Aliphatic Polyesters

##### Poly (Lactic Acid)

**Figure 8:**
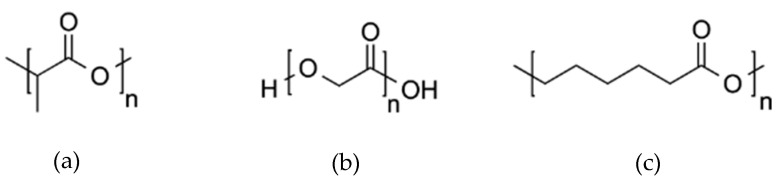
Chemical structure of (**a**) PLA, (**b**) PGA, and (**c**) PCL [82].

Lactic acid is produced naturally by plants, animals, and microorganisms via bacterial fermentation of starch or sugar. Production of polylactic acid (PLA) involves the dimerisation of two lactic acid monomers to form lactide, which is then polymerised via ring-opening polymerisation to form PLA. Different forms of PLA can be produced depending on the chirality of the base lactic acid monomer; poly-L-lactic acid (PLLA), poly-D-lactic acid (PDLA), and poly-D-L-lactic acid (PDLLA). PLA-based polymers have been employed in various applications, including drug delivery, fertilisers, and pesticides, as well as for bone fixation devices, packaging products, and interior automotive components [78]. The chemical structure of PLA is shown in Figure 8a.

##### Poly (Glycolic Acid)

Poly glycolic acid (PGA) is a biodegradable biopolymer synthesised from glycolic acid via polycondensation or glycolide via ring opening polymerisation. The commercial production of glycolic acid is currently still reliant on fossil fuel-derived compounds; however, alternative sustainable bio-chemical production pathways have been reported [79]. Much like polylactic acid, PGA has found many applications in the biomedical industry for absorbable sutures, dental implants, as well as orthopaedic and tissue scaffolds. The mechanical properties and degradability of PGA can be enhanced by co-polymerisation with PLA to form poly (lactic-co-glycolic acid) (PLGA) [80]. The chemical structure of PGA is shown in Figure 8b.

##### Poly (Caprolactone)

Polycaprolactone (PCL) is produced commercially via the ring-opening polymerisation of fossil fuel derived ε-caprolactone. Much like PLA and PGA, PCL has been extensively utilised in the biomedical field, and its slower degradation rate in vivo has led to its use in longer-term applications of up to 1–2 years [81]. The chemical structure of PCL is shown in Figure 8c.

### 3.4. Natural Polymers

#### 3.4.1. Microorganisms

##### Poly (hydroxyalkanoates)

**Figure 9:**
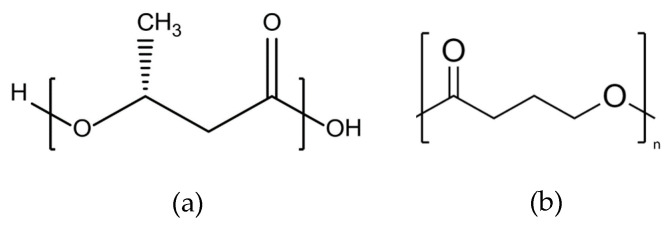
Chemical structures of (**a**) PHB [85] and (**b**) P4HB [86].

Poly (hydroxyalkanoate) is produced via bacterial fermentation of carbohydrate biomass and can be extracted from cells by the use of organic solvents. The extraction process results in the conversion of amorphous PHA to a highly crystalline and brittle form. The physical and chemical behaviour of PHA can be controlled by adjusting the fermentation feedstock to include valerate or butyrate compounds. This results in the formation of co-polymers such as poly (hydroxybutyrate) (PHB), poly (3-hydroxyvalerate) (PHV), and poly (3-hydroxybutyrate-co-3-hydroxyvalerate) (PHBHV) [83]. Due to their enhanced properties, PHA co-polymers have been identified for potential application in a variety of fields, including drug delivery, food packaging, water treatment, and polymer composites [84]. The chemical structure of PHB and P4HB is shown in Figure 9.

#### 3.4.2. Polysaccharides

Polysaccharides are a diverse group of long-chain carbohydrate polymers that are composed of monosaccharide units (sugars) held together by glycosidic bonds. Polysaccharides perform multiple functions throughout nature, including as structural elements and energy storage [72]. Due to their abundance and biocompatibility, polysaccharides have been used extensively in the food and cosmetics industries and have also been identified as potential feedstocks for the production of bio-based thermoplastic polymers.

##### Cellulose

Cellulose acetate (CA) is produced by the acetylation of cellulosic biomass, commonly obtained from wood pulp or cotton, and compounded with a plasticiser to enable melt processing which would be essential for production at industry scale. Additional modified cellulose esters that offer some variability in heat distortion temperature and water absorption are also available, such as cellulose acetate propionate (CAP), cellulose acetate butyrate (CAB), and cellulose propionate (CP) [87]. Indicative mechanical properties of various cellulose-derived polymers are shown below (Table 4).

##### Starch

Native starch is an abundant, renewable biopolymer composed of two distinct polysaccharides: amylose and amylopectin. Its primary function within plant cells is to provide a means of energy storage and can be obtained from annual crops such as wheat, corn, and potatoes. In its native form, starch degrades well below its melting temperature; however, thermoplastic derivatives can easily be obtained through the incorporation of plasticisers such as water, glycerol, or urea [91]. Although melt processable, thermoplastic starch (TPS) still lacks the necessary mechanical properties to be seriously considered for most commercial applications. Therefore, further modification via polymer blending or nanofillers is required to improve the overall performance of TPS materials. Current research largely focuses on the potential for TPS as a food packaging and agriculture material [92].

Esmaeili et al. [93] investigated the optimum ratio of sorbitol and glycerol for plasticising native starch. It was observed that a minimum plasticiser content of 36% was required to obtain melt-processable TPS. The incorporation of sorbitol was seen to have a greater enhancement effect than glycerol, exhibiting tensile strength and elastic modulus of approximately 24.0 MPa and 1.60 GPa, respectively. However, agglomeration occurred when the percentage of plasticiser was increased beyond 36.0%, degrading the mechanical properties.

##### Chitosan

Chitosan is the deacetylated form of chitin, the main constituent in the exoskeletons of crustaceans. It is a low-cost, biodegradable polymer with potential applications in the packaging industry and the biomedical field owing to its antibacterial properties. As in the case of TPS, chitosan requires the incorporation of plasticisers to improve its melt processability. Matet et al. [94] investigated the effect of glycerol, xylitol, and sorbitol on the mechanical and thermal properties of chitosan. All polyols had a strong plasticising effect on chitosan, increasing the tensile strain at break from approximately 30.0% for neat chitosan to 100%, 85.0%, and 80.0% for glycerol, xylitol, and sorbitol, respectively. This increase in elongation was accompanied by a decrease in tensile strength.

##### Seaweed

Recently, seaweed-derived biopolymers have attracted a substantial amount of attention as a potential bio-based resource for plastic production. Seaweed is a generic term given to multicellular macroalgal species that grow in coastal marine environments. Although they vary widely in structure, seaweeds are generally classified based on their colour, being either red, green, or brown. Alginate is a common seaweed-derived polysaccharide extracted from brown algae such as kelp. Its biocompatibility and gelling properties have led to its extensive use in pharmaceutical and biomedical applications [95]. Current research efforts aim to further extend the use of seaweed-derived biopolymers to commercial and packaging applications via various techniques, such as polymer blending [96].

### 3.5. Bio-Epoxy

Epoxy resins are thermosetting prepolymers capable of forming a rigid crosslinked network when cured with a hardener and/or heat. They can be classified based on their chemical precursors, with most commercially available resins being synthesised from fossil fuel-derived compounds like diglycidyl ether of bisphenol-A (DGEBA), a product of the reaction between bisphenol-A and epichlorohydrin. Bio-derived epichlorohydrin has already been utilised in commercial bio-based epoxy resins, being synthesised from glycerol rather than propylene [97]. Due to global concerns over depleting non-renewable resources, much attention has been paid in recent years to the identification of renewable precursor chemicals to replace DGEBA. The most promising candidates include epoxidised plant oils, saccharides, and polyphenolic compounds [98].

#### 3.5.1. Plant Oils

**Figure 10:**
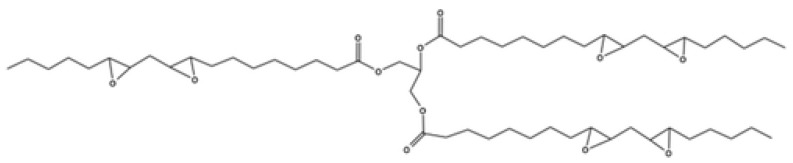
Chemical structure of epoxidised linolein, a major constituent of ESO [99].

Plant oils are an abundant natural feedstock extracted from seed and legume crops such as soy, flax, rapeseed, castor, and palm. Their basic structure is that of three fatty acids (triglyceride, see Figure 10), the components of which depend on the crop and variety. The triglyceride structure of plant oils is well suited for modification, possessing various active sites such as carbon/carbon double bonds and ester groups [99].

The epoxidation of the carbon/carbon double bonds is the most widely used modification method to achieve high molecular weight thermoset polymers and is carried out via simple acid ion exchange, using a carboxylic acid and hydrogen peroxide as the oxygen carrier and donor, respectively [100]. Modified plant oils have been employed as co-monomers to produce compound thermoset resins and as plasticisers to increase the bio-based content of existing commodity prepolymers [101].

##### Epoxidised Soybean Oil (ESO)

The vast majority of soybean oil is used in the food industry, with a small portion used for the production of biofuel and as a renewable substitute for petrochemicals in industrial applications [102]. This includes its use as a plasticiser in commodity polymers and as a precursor chemical in bio-epoxy resin production.

Partial bio-based epoxy resin systems were developed by Miyagawawa et al. [103] that substituted between 30.0 and 50.0 wt% of diglycidyl ether of bisphenol-F (DGEBF) with ESO, exhibiting enhanced impact strength and fracture toughness. Takahashi et al. [104] investigated the effect of various anhydride curing agents on the mechanical and thermal properties of ESO thermosets. The properties of the resultant cured thermosets greatly varied with the curing agent utilised, with terpene and hexahydropthalic cured thermosets exhibiting tensile strengths of 22.0 MPa and 15.0 MPa, respectively. Thermosets cured with maleinated linseed oil possessed a tensile modulus of approximately 24.0 MPa, compared to 755 MPa and 592 MPa for terpene and hexhydropthalic curing agents, respectively.

##### Epoxidised Linseed Oil (ELO)

Interest in the use of linseed oil in bio-epoxy resins has grown in recent years due to its high degree of unsaturation, allowing many epoxy groups to be grafted throughout its structure. The abundance of epoxide rings in ELO has made it an ideal candidate for the production of strong, highly crosslinking bio-epoxies [105]. Similar to ESO, ELO has been utilised as both a plasticiser and a direct replacement for fossil-based epoxy systems.

Sahoo, Khanelwal, and Manik [106] prepared partially bio-based epoxy resins by substituting between 0.00 and 30.0% DGEBA for ELO, utilising a bio-based phenalkamine curing agent. The addition of ELO had a plasticising effect, increasing the elongation at break from 3.50% to 13.5%. The impact strength of the samples also increased from 3.49 kJ/m^2^ to 4.99 kJ/m^2^ for the 30.0% mixtures. However, a reduction in both tensile strength and tensile modulus was also observed at the same ratio. Dominici et al. [107] developed ELO thermosets crosslinked with methyl nadic anhydride (MNA), exhibiting flexural strength and modulus of 60.8 MPa and 1.77 GPa.

##### Epoxidised Castor Oil (ECO)

Castor oil is a low-cost plant oil extracted from the seeds of the perennial castor plant. Major components of Castor oil include ricinoleic acid (90%), linoleic acid (4.2%), oleic acid (3%), stearic acid (1%), and palmitic acid (1%). Castor oil possesses good functionality due to the presence of one hydroxyl and one unsaturated group per fatty acid chain that can be readily epoxidised [108].

Paluvai, Mohanty, and Nayak [109] prepared blended DGEBA epoxy thermosets with varying portions of ECO from 0.00 to 40.0%. The addition of ECO had a plasticising effect, increasing the elongation at break from 5.70% for neat DGEBA epoxy up to 49.0%. A decrease in tensile strength and modulus was also observed. The plasticising effect was attributed to the flexibility of the long-chain fatty acid molecules, reducing the rigidity of the epoxy matrix. Partially bio-based ECO and DGEBA epoxy thermoset blends were also developed by Sahoo, Khanelwal, and Manik [110]. As the percentage of ECO increased from 0.00 to 30.0%, the tensile strength decreased from 55.0 MPa to 26.0 MPa, and the elongation at break increased from 3.50% to 6.20%. The addition of 30.0% ECO was also found to increase the impact strength of neat DGEBA by 146%.

#### 3.5.2. Saccharides

##### Furans

**Figure 11:**
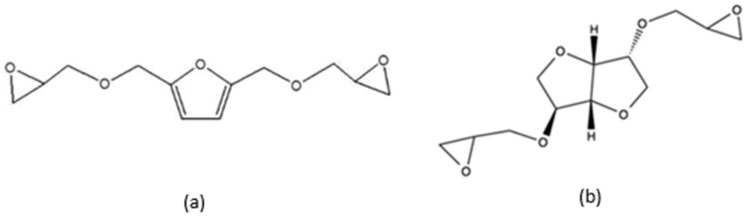
Chemical structure of (**a**) BOF and (**b**) DGEI [99].

Due to their rigid aromatic structure and their abundance in biomass feedstocks, furan-based compounds have been identified as a potential bio-based DGEBA replacement in epoxy resin systems [111]. 2,5-bis[(oxiran-2-ylmethoxyl) methyl] furan (BOF, see Figure 11a), difurfuryl amine, and furfuryl alcohol have all shown potential as bio-based monomers, curing agents, and additives, respectively.

Meng et al. [112] compared the mechanical and thermal properties of BOF to DGEBA resins cured with 4,4′-diamino diphenyl-sulfone (44DDS). DSC and DTG analysis revealed that bio-based thermosets possessed lower glass transition temperatures compared to DGEBA and were subject to partial degradation at low temperatures. However, the temperature at 30.0% decomposition (T_d30_) and at the maximum decomposition rate (T_max_) were both similar to that of DGEBA and also exhibited greater than 40.0% residual char, indicating inherent fire-retardant properties. The BOF/44DDS networks also displayed superior tensile (75.5 MPa) and flexural (91.8 MPa) strengths compared to DGEBA (44.6 MPa and 67.3 MPa, respectively).

##### Isosorbides

Isosorbide is a low-cost, rigid, thermally stable molecule extracted from starch crops. Isosorbide can be extracted from starch in a three-step process of hydrolysis to D-glucose, hydrogenation to sorbitol, and finally, dehydration to isosorbide. Due to its high rigidity and functionality, isosorbide is considered a promising candidate as a renewable replacement for bisphenol-A [113].

There are multiple ways in which isosorbide can be epoxidised into a suitable epoxy precursor chemical. The simplest method involved the direct reaction of isosorbide with epichlorohydrin to form diglycidyl ether of isosorbide (DGEI, see Figure 11b). Due to its similarities with the production of DGEBA, this reaction scheme presents the most industrially feasible method of producing isosorbide precursors [114].

Hong et al. [113] investigated the properties of DGEI resins cured with diethylene triamine (DETA) and isosorbide diamine (ISODA) hardeners. DGEI thermosets cured with DETA exhibited superior tensile strength and Young’s modulus as compared to commercial DGEBA polymers. The tensile properties were further enhanced when cured with a bio-based ISODA hardener. However, DGEI thermosets also exhibited much lower glass transition temperatures (50.0–52.0 °C) than DGEBA samples (130 °C).

#### 3.5.3. Polyphenols

Polyphenols are a diverse family of chemicals present throughout nature. They are characterised by the presence of hydroxyl groups bonded to aromatic phenyl rings [115]. Their rigid aromatic structure, abundance in nature, and diverse functionality make polyphenols a promising bio-based alternative to fossil fuel-derived epoxy monomers.

##### Lignin

Lignin is a complex three-dimensional polyphenolic molecule found in the cell walls of plant cells. Commercially, lignin is obtained as a by-product of the forestry and pulping industry and is commonly burned for the purpose of heat recovery [116]. As an abundant, aromatic, and complex renewable resource, lignin has attracted great interest for its potential applications in the chemical and polymer industries. Lignin can be utilised directly as a by-product from the delignification process, or it can be depolymerised and chemically modified to obtain other high-value products such as vanillin. Current research is largely focused on the extraction and purification of these high-value derivatives, many of which may have the capacity to replace fossil fuel-derived monomers [99].

Asada et al. [117] investigated the suitability of epoxidised, low molecular weight lignin as a replacement for commercial DGEBA resins in the manufacture of printed circuit boards (PCB). Although exhibiting lower decomposition temperatures than DGEBA thermosets, the epoxidised lignin thermosets all satisfied the solder-dip resistance test, indicating they could be suitable in electronics applications. Fache et al. [118] prepared several vanillin-based epoxy resins that were subsequently crosslinked with an amine-curing agent (IPDA). The bio-based thermosets exhibited glass transition temperatures, storage moduli, and temperatures of maximum degradation that were comparable with DGEBA networks.

##### Gallic Acid

Gallic acid is a naturally occurring phenolic acid that possesses three phenolic hydroxyls and one carboxyl group. Traditional epoxidisation methods that involve the direct reaction of gallic acid with epichlorohydrin often lead to incomplete functionalisation. However, novel two-step preparation methods have also been developed to avoid this [119]. These methods involve the initial functionalisation of phenolic hydroxyl and carboxyl groups prior to epoxidisation to form usable chemicals, such as glycidyl ether of gallic acid (GEGA).

Aouf et al. [119] compared the thermomechanical properties of GEGA thermosets cured with a diamine hardener (IPDA) to those of DGEBA. Analysis revealed an increase in glass transition temperature, indicating higher crosslinking densities. This was attributed to the increased functionality of gallic acid as compared to the DGEBA. Tarzia et al. [120] similarly attributed the superior tensile properties of GEGA/IPDA thermosets, as compared to DGEBA-based networks, to the increased functionality and resultant higher crosslink density of gallic acid.

### 3.6. Properties of Biopolymers

#### 3.6.1. Mechanical Properties

The tensile and flexural properties of various thermoplastic biopolymers and bio-based epoxy resins are shown in Table 5 and Table 6. The results in Table 5 show the wide range of tensile strengths for injection moulded samples, from as low as 22.0 MPa for PHBHV up to 74.4 MPa and 141 MPa for PLA and PGA, respectively. Most polymers exhibit brittle behaviour, possessing values of elongation at break less than 10.0%. When compared to the mechanical properties of common fossil fuel-derived thermoplastics such as polypropylene (PP), low-density polyethylene (LDPE) and polyvinyl chloride (PVC), the properties of most biopolymers can be seen to be similar, or in the case of PLA, PGA, CA, and CAP, exceed both their tensile strength and modulus. The tensile strengths of thermoset biopolymers in Table 6 are all lower than epoxy and polyurethane thermosets, except in the case of DGEI and BOF, respectively. In addition, GEGA thermosets display a tensile strength that is similar in magnitude to epoxy, with a greater modulus. This highlights the advantages of utilising naturally aromatic compounds in the synthesis of bio-based thermosets compared to aliphatic compounds such as epoxidised plant oils.

#### 3.6.2. Thermal Properties

The thermal properties of various thermoplastic and thermoset biopolymers are shown in Table 7 and Table 8, respectively. The results in Table 7 show a wide range of thermal properties available for thermoplastic biopolymers. The low glass transition temperature (T_g_) of PCL offers some explanation as to the elastic behaviour discussed previously, with large elongation at break attributable to increased chain mobility. The melting temperature (T_m_) range of biopolymers such as PLLA, PLA, and PHB are very similar to existing fossil fuel-derived polymers such as polypropylene [126], making them attractive alternatives to incorporate into existing technical applications and can be processed using existing manufacturing facilities. Conversely, excluding GEGA, the glass transition temperatures of most thermoset biopolymers shown in Table 8 are much lower compared to epoxy systems [127] and exhibit lower temperatures of 5.00 and 10.0% weight loss (i.e., T_d5_ and T_d10_, respectively).

### 3.7. Limitations of Bio-Based Polymers

Biopolymers have the potential to reduce the environmental impacts associated with plastic waste and decrease global reliance on fossil fuel resources. However, several key limitations of biopolymers must first be addressed if they are to be utilised in wider applications. These include their often inferior mechanical and thermal performance, poor environmental durability, high cost, and limited availability.

#### 3.7.1. Performance

Around 40.0% of all plastics produced globally are for use in packaging applications. Two thirds of this supply can be associated with food and beverage products, with the remainder being used in medical, consumer, household items, and shipping. A range of thermoplastic polymers are currently employed as packaging materials. These include polyethylene terephthalate (PET), polypropylene (PP), polystyrene (PS), expanded polystyrene (EPS), low-density polyethylene (LDPE), and high-density polyethylene (HDPE) [129].

Starch and cellulose-based films have been developed for food packaging applications; however, neat compositions often exhibit poor tensile strength and brittle behaviour. Moreover, their comparatively poor thermal processability prevents them from being easily integrated into existing thermoforming manufacturing processes. Due to their hydrophilic nature, starch and cellulose-based packaging films also exhibit low moisture resistance and vapour barrier properties [130].

Common thermoplastic polymers such as acrylonitrile butadiene styrene (ABS), polyamide (PA), polycarbonate (PC), PP, and LD/HDPE are used to produce a range of automotive components, including interior door panels, dashboards, centre consoles, instrument panels, bumpers, seating, trim, fuel systems, liquid reservoirs, electrical systems, engine covers, and cooling systems. Although material selection criteria in automotive design vary based on the desired functionality, each component is generally expected to possess adequate tensile and flexural properties, wear and abrasion resistance, and good thermal stability. Additional requirements related to impact strength also apply in the case of exterior body panels [131]. Several commercially available biopolymers, such as PLA, PHA, and PBS, exhibit mechanical properties similar to fossil fuel-derived polymers; however, uncertainty regarding their long-term durability still limits their use in most applications without additional modification and enhancement.

#### 3.7.2. Environmental Durability

Biodegradable biopolymers are inherently less resistant to UV radiation, microbiological attack, and hydrolysis as compared to non-biodegradable polymers. These attributes are advantageous in the context of biodegradability and compostability; however, they also threaten to limit the use of biopolymers to short life cycle applications if degradation rates cannot be suitably controlled [132].

Exposure to elevated temperatures in the range of 50.0–70.0 °C for prolonged periods has the potential to cause heat distortion effects, a reduction in mechanical properties, and thermal degradation. Alternatively, exposure to sub-zero temperatures in arctic climates could embrittle bio-based components, increasing the likelihood they will fail prematurely [133]. Quispe, Lopez, and Villar [132] investigated the photo-degradation of TPS by exposure to UV radiation. Following 264 h of exposure, the TPS microstructure exhibited signs of degradation in the form of cracks and pores, and samples were noted to be extremely brittle, as confirmed by an observed 85.0% reduction in their elongation at break.

#### 3.7.3. Cost and Availability

Due to the limited scale of global production as compared with traditional polymers and additional research and development expenses, the cost of commercially available biopolymers is currently (2024) between 2 and 10 times greater than fossil-based polymers. This price gap is a significant limiting factor in their widespread adoption in many applications [134]. Starch-based polymer blends, PLA, and PBAT, are among the most widely used biopolymers currently in the market, with applications largely confined to biodegradable packaging materials. Although substantial growth is expected within this sector, biopolymers still represent a very small portion of the global plastic sector [135].

### 3.8. Enhancement Strategies

#### 3.8.1. Alternative Synthesis Routes

In an effort to reduce the cost and environmental impacts associated with biopolymer production, alternative synthesis routes have been proposed that utilise novel feedstocks such as gaseous carbon or complex carbon sources from industrial waste streams. Chavez, Raghaven, and Tartakovsky [136] reviewed several alternative PHA production processes and identified the most important factors affecting production costs were carbon source selection, yield, and productivity. Production processes that utilised alternative carbon sources from industrial waste streams had lower production costs; however, there was a much larger uncertainty associated with them due to the additional purification requirements. Production costs were further reduced by claiming environmental credits for diverting industrial waste from treatment plants and for the generation of hydrogen as a by-product.

#### 3.8.2. Polymer Blending

A polymer blend is defined as a mixture of two or more polymers or copolymers. Polymer blending offers manufacturers an opportunity to modify the properties of a biopolymer to suit a given target application, often with a reduction in overall material costs. Blending can be achieved using various manufacturing methods, including melt blending, solution blending, copolymerisation approaches, and interpenetrating polymer networks. Due to the poor miscibility of highly polar biopolymers, it is often necessary to add compatibilisers to increase interfacial adhesion and ensure their complete incorporation [137].

Fourati et al. [138] investigated the effect of various compatibilisers on the mechanical properties of PBAT/TPS polymer blends compounded via extrusion at a 60/40 weight ratio. The introduction of 2.00% malleated PBAT to the blend greatly improved the interfacial interactions between PBAT and TPS phases, resulting in an increase in tensile strength, modulus, and elongation at break. Gong, Qiang, and Ren [139] demonstrated a one-step reactive extrusion method for toughening PLA through the inclusion of PHBV at a weight ratio of 80/20 and the addition of 0.3 wt% peroxide initiator (DBPH). The addition of 0.1 wt% peroxide initiator increased both tensile strength and elongation at break of the blends due to improved compatibility in PLA and PBAT phases.

#### 3.8.3. Reinforcement

Reinforcement with fibres or nanofillers is a cost-effective method for improving the performance and versatility of biopolymers. Various nanofillers have been investigated in recent years, including silicate/non-silicate minerals, polysaccharides, carbonaceous matter, and metal oxides [140]. Famá et al. [141] developed starch-based/multi-walled carbon nanotube (MWCNT) nanocomposites that exhibited a 35.0% increase in tensile strength and a 70.0% percent increase in Young’s modulus with just 0.055 wt% filler content. Similarly, natural fibres have been identified as a cost-effective and renewable reinforcement material to improve the properties of biopolymers, making them suitable for a wider range of applications [142].

### 3.9. Summary

In summary, biopolymers are derived from bio-based natural resources and/or are biodegradable. Research into biopolymers has greatly increased in recent years due to the co-ordinated effort to reduce global reliance on fossil fuels and limit plastic pollution. Both thermoplastic and thermosetting biopolymer materials have been synthesised from fossil fuel sources, microorganisms, polysaccharides, plant oils, and polyphenolic compounds. Several factors currently limit the widespread use of biopolymers in most industrial and commercial applications, including their largely inferior mechanical and thermal properties, poor environmental durability, high cost, and limited availability. Various enhancement strategies have been proposed to address these limitations, such as using alternative synthesis routes and waste feedstocks, blending with other polymers, and reinforcing with bio-based nano-fillers or fibres.

## 4. Bio-Composites

### 4.1. Manufacturing of Bio-Composites

There are a variety of fabrication techniques available for the preparation of composites, including hand lay-up or spray-up, resin transfer moulding (RTM), vacuum-assisted resin transfer moulding (VaRTM), injection moulding, or compression moulding [143]. For natural fibre-reinforced composites, the temperature at which they are processed is an important consideration to avoid thermal degradation of the fibres. Hemicellulose is the least stable natural fibre constituent, degrading between 200 and 260 °C, followed by cellulose, which decomposes between 240 and 350 °C and finally, lignin in the range of 280–500 °C [58]. It is therefore important that natural fibre-reinforced composites are processed at temperatures below the lowest onset decomposition temperature, i.e., 200 °C. Thomason and Ruderios-Fernandez [144] investigated the impact of heat treatment on the mechanical properties of coconut coir. Results indicated that heat treatment in air at 200 °C for 30 min reduces the tensile strength of the fibres by approximately 30%. This was attributed to the decomposition of less thermally stable compounds such as hemicelluloses and pectin found in the secondary cell wall, reducing the fibre’s ability to sustain plastic deformation.

The inherent moisture content in natural fibres can also affect the mechanical properties of a bio-composite if not sufficiently dried prior to fabrication. Todorvik et al. [145] found that the moisture content of flax fibres (FF) strongly affected the performance of FF/bio-based epoxidised linseed oil composites. It was observed that the presence of water inhibited chain cross-linking during the curing process through hydrolysis of the ELO epoxide groups, as evidenced by a decrease in glass transition temperature. Additionally, the vaporisation of excess water during the de-gassing and curing processes led to the formation of voids at the fibre/matrix interface and resulted in poor mechanical performance compared to dried fibre composites.

### 4.2. Properties of Bio-Composites

#### 4.2.1. Mechanical Properties

The tensile properties of various flax fibre composites are shown in Table 9 below. The tensile strength and modulus of randomly oriented flax/PLA and flax/PP composites are comparable, with PLA composites being slightly superior to both PP and other biopolymer matrices such as PHB. Notably, flax fibre-reinforced CAB composites possess equivalent tensile strength to RM0 flax/PLA, with a slightly reduced stiffness. For unidirectional and aligned arrangements, flax/PLA composites exhibit tensile strength and Young’s modulus of 177 MPa and 10.8 GPa versus 143 MPa and 7.34 GPa for flax/PP composites. The performance of unidirectional bio-epoxy composites is also close to that of flax/epoxy composites, exhibiting tensile strengths and Young’s moduli of 360 MPa and 28.7 GPa, and 339 MPa and 33.3 GPa, respectively. The comparable properties of PLA and bio-epoxy composites with their synthetic matrix counterparts demonstrate the feasibility of utilising bio-based polymers in flax bio-composites.

#### 4.2.2. Thermal Properties

A summary of the thermal properties of a variety of bio-composites is shown in Table 10. The glass transition temperature (T_g_) of all composites is slightly higher than those of neat polymers due to the flax fibres inhibiting chain mobility [157]. In flax/PLA and flax/PCL composites, the effect is very slight, with only a 2.00 °C difference in either case. However, for the flax/PHB composite, the difference is more pronounced, with an increase of around 12.0 °C. The degree of crystallinity obtained via DSC was found to be slightly higher when compared with theoretical values calculated from composite mass fraction, suggesting that flax fibres have also acted as nucleating agents for crystal growth [158].

The thermal degradation behaviour of various bio-composites is shown in Table 11. For flax/thermoplastic starch (TPS) composites, the addition of flax fibres results in an increase in thermal stability, as evidenced by the increase in temperature at 10% and 50% weight loss (T_d10_ and T_d50_, respectively) with increasing fibre content. This is attributable to the better thermal stability of flax fibre as compared to starch. Conversely, for flax/ELO composites, the addition of flax fibre resulted in a decrease in T_d5_ by 5.00 °C due to the poorer thermal stability of flax fibre compared to epoxidised linseed oil (ELO).

### 4.3. Limitations

Most limitations currently associated with the use of plant fibres in bio-composites are largely attributable to the inherent properties of the fibres themselves, such as variability [5], hydrophilicity [7], and low thermal stability [8]. Poor compatibility with polymer matrices due to fibre anisotropy and surface chemistry must also be considered [6].

#### 4.3.1. Fibre/Matrix Compatibility

Interfacial interaction between fibre and polymer matrix is an important determining factor in the optimisation of composite performance. Both chemical and physical interactions contribute to the establishment of an effective interphase region responsible for transferring applied loads from matrix to fibre. The most generally accepted measure of fibre/matrix adhesion is known as the interfacial shear strength (IFSS). Multiple experimental techniques have been developed to measure the IFSS, including the single fibre pull-out test, microbond test, push-out test, and single fibre fragmentation test [162]. A summary of IFSS observed for various flax fibre composites is shown in Table 12 below.

Interfacial strength for natural fibres is generally considered poorer than that of glass due to chemical incompatibilities between matrix and fibre; however, there are other factors that must also be considered. Thomason [166] observed the IFSS in jute/polypropylene composites to be much less than that of glass, carbon, and aramid fibres. This was attributed to the anisotropic nature of the jute fibres themselves, with differences in transverse and longitudinal coefficients of thermal expansion resulting in reduced residual stress at the fibre/matrix interface.

Baley et al. [165] investigated the effect of various chemical treatment methods on the surface energy of flax fibre and IFSS in flax/unsaturated polyester composites. Unlike in synthetic fibres, no correlation was observed between changes to the surface energy of flax fibres as a result of chemical modification and the IFSS of composite samples. SEM images of failed samples also revealed a complex damage mechanism, with evidence of crack propagation through the matrix material, along the fibre/matrix interface, the middle lamella of fibre bundles and through elementary fibres themselves. This unique failure mechanism of natural fibre composites highlights an important characteristic related to the inherent structure of plant fibres that must be considered when examining interfacial interactions. Similarly, Le Duigou et al. [167] observed two distinct failure mechanisms during microbond testing of flax/PLLA samples. The first is a conventional interfacial debonding, and the second is initiated by the failure of the interlayer ‘peeling’ effect within elementary fibres. The latter failure mechanism occurred in 18% of tested samples, demonstrating the importance of considering the role of interlayer adhesion in interfacial interactions between natural fibres and polymer matrices.

#### 4.3.2. Fibre Variability

As previously discussed, several factors related to climatic conditions, agricultural practises and processing techniques can contribute to large amounts of variability in the properties of flax fibres extracted from a given crop. Differences in fibre strength due to varietal and climatic changes have been shown to also correspond with differences in the properties of manufactured composites [48]. Similarly, structural defects such as kink bands introduced as a result of standard processing methods can lead to increased voids and stress concentrations within flax fibre composites, as evidenced by a large scattering in their reported tensile strengths [5].

#### 4.3.3. Anisotropy

Large differences between transverse and longitudinal properties of plant fibres arising from their microstructure have been shown to affect the mechanical performance of bio-composites [6]. This is due to the role of the transverse coefficient of thermal expansion in interfacial adhesion, as well as the importance of transverse properties in short chopped, randomly oriented composites. The transverse properties of jute fibres have been previously estimated [168] using a combination of off-axis thermomechanical testing and semi-empirical relationships derived from micro-mechanical models. The longitudinal stiffness at 25.0 °C was found to be 39.4 GPa, whereas the transverse stiffness at the same temperature was 5.50 GPa. The coefficient of thermal expansion of jute fibre was also investigated and found to be negative in the longitudinal direction with an average value of −15.0 µm/m·°C obtained over a temperature range of 25.0 to 95.0 °C. The coefficient of thermal expansion in the transverse direction was found to be much larger, with a value of 77.2 µm/m·°C.

Thomason [166] modelled the residual radial compressive stress of jute fibre-reinforced polypropylene composites and found it to be significantly less than glass, carbon, and aramid systems, aligning with the accepted view of interfacial bonding for fibre-reinforced composites. Failure stress was also modelled for both pure and maleic anhydride-modified polypropylene composites using values of interfacial shear strength calculated from residual stress results and was found to be in good agreement with experimental values. The consistent lack of performance for injected moulded natural fibre composites in the literature was thus attributed to the disparity between longitudinal and transverse/shear moduli. Furthermore, the thermo-mechanical anisotropy of natural fibres was found to be directly related to the low residual compressive stresses at the fibre/matrix interface, potentially explaining the limited stress-transfer capability commonly observed in natural fibre composites.

#### 4.3.4. Moisture Absorption

Natural fibres are hydrophilic, meaning they will readily absorb moisture over time due to the presence of hydroxyl groups in cellular compounds. Water molecules form hydrogen bonds with the natural fibres, disrupting the interfacial interactions and inhibiting the effective stress transfer from matrix to fibres [169]. In addition, micro-cracks can be developed due to differential swelling of the fibres and matrix that can lead to debonding and eventual delamination in composites.

Numerous studies have characterised the deteriorating effects of water absorption on the reinforcing effects of natural fibres, and it remains a significant limiting factor in the widespread adoption of bio-composites in structural applications. Assarar et al. [170] compared the moisture absorption behaviour of epoxy composites reinforced with glass and flax fibres. Flax/fibre composites had a saturated weight gain 12 times higher than glass/fibre, with greater degradation in tensile properties also observed in flax/fibre composites.

#### 4.3.5. Flammability

Natural fibres are composed of dried organic matter and are therefore highly flammable. This has the potential to limit their use in aerospace, transportation, electronics, and construction applications due to stringent regulatory requirements. The thermal degradation of cellulose, the main constituent in plant fibre cells, is a multi-stage process that occurs within the range of 50.0–340 °C involving the evaporation of absorbed moisture, the formation of intermediate pyrolysis compounds, and the release of gaseous products and volatiles such as CO, ethylene, methane, and alcohols [171].

The inclusion of natural fibres in polymer matrices has been shown to result in shorter ignition times, greater amounts of heat being released, and significant structural deformation as compared to neat polymer samples and glass fibre-reinforced composites [172]. As such, the addition or modification of natural fibre composites with flame retardant compounds is essential to limit their flammability to acceptable levels in line with industrial standards.

### 4.4. Enhancement Strategies

**Figure 12:**
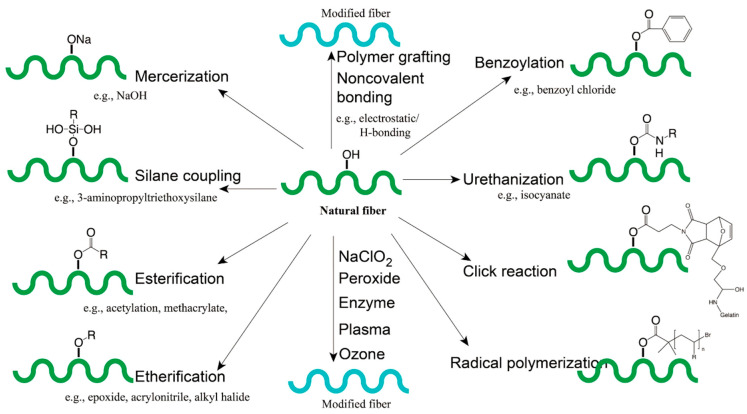
Chemical and physical modification of natural fibres [173].

Currently, research on natural fibre-reinforced composites often focuses on the effective compatibilisation of fibre and polymer matrices using chemical/physical treatment or fibre surface coatings. Moreover, the effects of fibre treatment in overcoming limitations of moisture absorption behaviour and flammability are also commonly investigated. A summary of chemical and physical modification strategies is shown in Figure 12.

#### 4.4.1. Chemical Treatment

The removal of impurities such as wax, pectin, and hemicellulose within raw fibres via chemical treatment enhances interfacial adhesion by (1) increasing the relative surface roughness, enabling strong mechanical interlocking, and (2) increasing the number of functional groups available for crosslinking with polymer matrix chains [174]. However, harsh chemical treatments also have the potential to reduce the effectiveness of natural fibres as reinforcing agents by degrading key microstructural components within plant fibres.

##### Alkalisation

The chemical treatment most employed in the preparation of flax/fibre composites uses an alkali solution, usually sodium hydroxide (NaOH), to dissolve non-cellulosic material and roughen the fibre surface. By decreasing the amount of hydrophilic material present within the fibre, moisture absorption behaviour can also be enhanced by alkali treatment [175]. At excessive concentrations and reaction times, treatment can also lead to a degradation of cellulose [176]. The optimal treatment parameters and the resulting change in mechanical properties for several composites reported in the literature are given in Table 13. The optimum treatment parameters that provided the largest increase in mechanical properties in the composites were a 5% NaOH solution carried out at ambient conditions for two hours.

##### Silane

Silane coupling agents have long been utilised to improve interfacial interactions and mechanical properties in glass fibre-reinforced composites, and similar improvements have been reported for natural fibres [180]. Hydrogen or covalent bond networks formed between hydrolysed silanol and the abundant hydroxyl groups of flax have been shown to improve the fibre’s compatibility with polymer matrices, resulting in enhanced mechanical properties [181] and moisture absorption resistance [182].

Le Moigne et al. [183] investigated the effect of organosilane fibre treatment on the mechanical properties of flax/PLA composites. Silane-treated flax composites exhibited a 20% and 4% increase in yield stress and impact strength, respectively, compared to untreated composites. Furthermore, yield stress and impact strength were further enhanced to 25% and 12% when an alkaline pre-treatment was utilised. The flexural properties of flax/PLA composites were also shown to be enhanced by the use of silane-treated fibres.

##### Acetylation

Acetylation is an esterification process that involves the introduction of acetyl groups into the molecular structure of a compound. In the case of natural fibres, this is usually carried out via treatment with acetic anhydride, during which the hydroxyl groups (OH) of the cell wall are substituted with acetyl groups (CH_3_CO) and acetic acid is generated as a byproduct. Acetylation is known to have a plasticising effect on cellulose, and the substitution of hydroxyl with acetyl functional groups also makes the treated natural fibres more hydrophobic [10].

Tserki et al. [184] investigated the effect of acetylation on the properties of flax fibre/polyester bio-composites. Tensile strength increased from 25.4 MPa to 28.6 MPa for treated samples, with an increase in Young’s modulus also observed. Scanning electron microscope (SEM) images of the fracture surfaces also revealed a difference in fibre/matrix interaction, as evidenced by less fibre pullout. Treatment with acetic anhydride also improved the moisture absorption resistance of the bio-composites, decreasing the saturated weight gain from approximately 6.5% to 4%.

#### 4.4.2. Physical Treatment

##### Plasma

Plasma treatment has been identified as a potential low-cost, rapid surface treatment method for improving fibre/matrix interactions without the need for chemicals. During treatment, natural fibres are briefly exposed to a plasma source containing ionised gas that removes impurities and activates the surface of the fibres. Enciso et al. [185] optimised the treatment of flax fibres via low-pressure plasma method to enhance their wettability. Contact angle measurements between flax fibre and distilled water showed a significant improvement in fibre wettability, decreasing from 121° for untreated fibre to 50.6° for a fibre treated for sixty seconds at 30 W in ionised air. Treated flax/LDPE composites exhibited enhanced mechanical properties, increasing the tensile strength and Young’s modulus of composites by 18.6% and 32%, respectively.

#### 4.4.3. Surface Coating

##### Polydopamine

Polydopamine (PDA) has been effectively utilised as a compatibiliser in natural fibre-reinforced composites due to its ability to adhere to both hydrophilic and hydrophobic surfaces. Bourmaud et al. [186] found that the mechanical properties of PLLA/hemp composites were improved when polydopamine was applied to the surface of PLLA pellets. Surface energy measurements and SEM revealed that the increase in tensile strength and modulus was attributable to the improved compatibility of the fibre and PDA-coated PLLA.

Zhang et al. [187] studied the influence of polydopamine and silane surface modification on the reinforcing ability of bamboo fibres in PLA composites. Polydopamine was found to effectively functionalise the fibre surface, providing an abundance of hydroxyl groups onto which silane may be grafted. The resultant modified bamboo/PLA composite was reported to possess a strong covalent bonded interfacial network that enhanced the mechanical properties and thermal stability compared to neat PLA and conventional silane-modified bamboo fibre composites.

Polydopamine has also been investigated as an effective bio-based fire retardant. Oktem and Aydas [188] reported an increased fire resistance in PDA-coated jute fabric and their corresponding epoxy composites, with both exhibiting delayed ignition time and decreased flame propagation rate. Similar improvements have been observed for polydopamine-coated flax fibre/PU composites. The fire-retardant properties of PDA were attributed to the protective char layer formed upon its decomposition that limited the fibre’s interaction with the heat, oxygen, and other flammable gases produced during combustion [189]. Zhang et al. [190] successfully improved the flame retardancy of flax/PLA composites through fibre surface modification by polydopamine and iron phosphonate. Peak heat release rate and total smoke production were both decreased in surface-modified fibre composites. The surface modification was also found to increase the tensile modulus of the resultant composites compared to neat PLA.

### 4.5. Summary

In summary, bio-composites are a class of composites that utilise bio-based materials in their preparation in an effort to reduce the environmental impact associated with traditional synthetic fibres and fossil fuel-derived polymers. Natural fibres, especially bast fibres such as flax, have been identified as a potential replacement for glass fibre in polymer composites due to their high specific properties. However, several limitations associated with the use of natural fibres must first be addressed if they are to be considered as a replacement for glass fibre. These include poor compatibility with polymer matrices, variability, high moisture absorption, and flammability. Various chemical, physical, and surface treatments have been investigated to tackle these drawbacks, such as alkalisation, silane treatment, acetylation, plasma treatment, and polydopamine surface treatment.

## 5. Conclusions and Future Perspectives

This review aims to evaluate the state of knowledge on the processing, properties, and limitations of natural fibres and biopolymers in the context of bio-composite materials. It also aims to give an overview of recent advancements and future outlooks for several enhancement strategies to overcome limitations in mechanical performance, moisture absorption, and flammability. From the review, the following conclusions can be drawn, and future work can be proposed:

- Flax fibres present as the most promising bio-based alternative to glass fibres owing to their high specific tensile properties and low cost. However, several limitations associated with variability and the development of critical defects remain inherent to the cultivation and processing methods used for fibre extraction. As such, the complex hierarchical microstructure of flax fibre and its unique interaction with biopolymer matrices must be carefully considered during the development and design of bio-composite materials.
- The inherently poor environmental durability of biopolymers, although advantageous in the context of disposal, also threatens to limit their use to short life cycle applications if this property cannot be accurately controlled. The environmental durability of novel thermosetting biopolymers (e.g., BOF, DEGI, and GEGA) that display comparable thermal and mechanical properties with existing fossil fuel-derived polymers should therefore be assessed to determine their suitability for long-term automotive, commercial, and aerospace applications.
- Although they address the concerns of environmental impact, bio-composites that utilise natural fibres and biopolymers still possess serval limitations that must be addressed if they are to be considered mainstream reinforcement and matrix materials, respectively. These include poor fibre/matrix compatibility, variability, high moisture absorption, and flammability. Numerous chemical, physical, and surface treatments have been examined to tackle these drawbacks; however, improvements have thus far been marginal. It is therefore recommended that research efforts be largely directed towards the development of novel fibre treatment methods using bio-based compounds with inherent hydrophobic and flame-retardant properties, such as phosphates, furans, or certain polysaccharides. This will allow for the issues of fibre/matrix compatibility, moisture absorption, and flammability to be tackled concurrently, expediting the development of high-performance natural fibre bio-composites for wider applications.

## Figures and Tables

**Table 1:** Economic, physical, and mechanical properties of plant fibres by type. Average costs as of ^a^ 2018, ^b^ 2017, ^c^ 2007, and ^d^ 2020.

Type	Fibre	Average Cost ($/kg)	Density (kg/m^3^)	Tensile Strength (MPa)	Young’s Modulus (GPa)	Elongation at Break (%)	Ref.
Bast	Flax	3.15 ^a^	1.33–1.65	340–1500	18.0–80.0	1.20–3.20	[11,12,13,14,15,16,17]
	Hemp	1.55 ^a^	1.47–1.57	550–900	6.00–70.0	1.60–4.70	[12,13,14,15,16,17]
	Jute	0.950 ^a^	1.30–1.50	320–800	10.0–30.0	1.00–1.80	[12,13,14,15,16,17,18,19]
	Kenaf	0.400 ^a^	0.749–1.45	223–930	1.86–53.0	1.50–6.90	[12,13,14,15,19]
	Ramie	2.00 ^b^	1.30–1.80	400–1100	44.0–130	1.20–4.00	[13,14,15,16,17]
	Nettle	-	1.50	650–1590	38.0–87.0	1.00–6.00	[12,14]
Leaf	Sisal	0.650 ^a^	1.27–1.50	400–800	9.00–38.0	2.00–14.0	[12,13,14,15,16,17,19]
	Abaca	0.345 ^b^	1.50	400–980	12.0–33.6	2.90–10.0	[12,14,15]
	Henequen	-	1.20	500	13.2	4.80	[12,14]
	Fique	1.01 ^d^	0.970–1.33	411–509	8.60–8.73	4.90–9.40	[20,21]
	Pineapple	0.455 ^b^	1.20–1.50	170–627	12.0–82.0	1.00–4.80	[12,15,16]
	Banana	0.890 ^c^	0.750–1.50	355–914	7.70–32.0	1.50–5.30	[12,13,14,15,16]
Grass	Bamboo	0.500 ^a^	0.600–1.10	140–800	5.96–36.0	1.30–10.4	[12,14,15,19]
	Bagasse	-	1.20	20.0–290	19.7–27.1	1.10	[13,14,15]
	Rice straw	-	0.900–1.50	100–200	0.300–12.5	5.40–10.6	[12,15]
	Wheat straw	-	1.10–1.49	59.0–150	3.70–4.80	3.50–6.60	[12,15]
Seed	Cotton	2.85 ^a^	1.30–1.90	287–597	5.10–13.0	2.00–10.0	[12,13,14,16,17]
	Coir	0.320 ^a^	1.15–1.50	106–593	1.27–6.00	15.0–47.0	[12,13,14,15,16,17,18]

**Table 2:** Details of flax fibre origin and retting method.

Variety	Retting Method	Fibre Extraction Method	Location	Diameter (um)	Ref.
Hermes	-	Mechanical	-	16.0 ± 1.70	[5]
Adriene (Dried)	Dew retted	Mechanical	France	21.6 ± 0.950	[53]
Adriene (Undried)	Dew retted	Mechanical	France	23.9 ± 0.680	
-	Green	Hand	Poland	18.9 ± 4.30	[47]
-	Dew retted	Cottonised	Poland	18.4 ± 3.00	
-	Dew retted	Mechanical	Poland	-	[52]
Diana	Dew retted	Mechanical	Latvia	-	
Marylin	Dew retted	Mechanical	France	13.6 ± 2.50	[54]
Hermes	Dew retted	Mechanical	France	21.0 ± 7.00	
Oliver	Dew retted	Mechanical	France	17.0 ± 3.70	
Hivernal	Dew retted	-	France	13.3 ± 5.20	[55]
Niagra	Dew retted	-	France	15.3 ± 5.20	
Alaska	Dew retted	-	France	13.8 ± 4.70	
Eden	-	-	France	15.2 ± 2.60	[56]
Alize	-	-	France	16.3 ± 4.80	

**Table 3:** Thermal properties of neat and treated flax fibres.

Details	Stg 1		Stg 2		Carbon Residue (%)	Ref.
	T_max_ (°C)	Wt. Loss (%)	T_max_ (°C)	Wt. Loss (%)		
Untreated	360	49.0	600	15.3	-	[59]
Alkaline treated	360	54.4	605	18.8	-	
Retted	334	68.7	432	24.3	0.800	[60]
Unretted	341	54.6	445	31.4	9.32	[61]
Enzyme retted	354	62.9	440	27.2	6.24	
Water Retted	355	63.3	456	26.4	6.50	
Untreated	348	58.0	483	38.0	-	[62]
Alkaline treated	344	69.0	445	29.0	-	
Enzyme treated	352	76.0	466	18.0	-	
Steam-heated	350	61.0	454	34.0	-	

**Table 4:** Tensile properties of cellulose-derived polymers.

Cellulose Derivative	Plasticiser Content	Young’s Modulus (GPa)	Tensile Yield Stress (MPa)	Elongation at Break (%)	Ref.
Cellulose Acetate	20.0%	3.10 ± 0.100	58.3 ± 4.70	8.30 ± 3.10	[88]
Cellulose Acetate Propionate	12.0%	-	33.0	27.0	[89]
Cellulose Acetate Butyrate	0.00%	0.341 ± 0.0147	18.0 ± 1.40	4.10 ± 0.390	[90]
Cellulose Propionate	7.00%	-	35.0	60.0	[87]

**Table 5:** Mechanical properties of injection moulded thermoplastic biopolymers and common fossil derived thermoplastics.

Polymer	Tensile Strength (MPa)	Young’s Modulus (GPa)	Elongation at Break (%)	Flexural Strength (MPa)	Flexural Modulus (GPa)	Impact Strength (kJ/m^2^)	Ref.
PLA	74.4 ± 0.200	3.44 ± 0.0860	7.8 ± 2.00	108 ± 0.600	3.20 ± 0.0340	2.10 ± 0.600	[121]
PGA	141 ± 3.00	7.31 ± 0.353	2.5 ± 0.400	217 ± 4.80	6.74 ± 0.236	2.30 ± 0.800	
PCL	30.9 ± 1.40	0.445 ± 0.00700	1250 ± 103	22.4 ± 0.100	0.430 ± 0.0190	15.5 ± 0.600	
PHB	40.0	3.50	6.00	-	-	-	[122]
PHBHV	22.0 ± 0.480	0.889 ± 0.0410	9.90 ± 1.10	-	-	-	[123]
CA	44.2 ± 0.600	4.39 ± 0.106	4.30 ± 0.500	69.4 ± 1.10	4.55 ± 0.149	1.80 ± 0.00	[121]
CAP	58.2 ± 5.00	2.48 ± 0.0220	3.00 ± 0.600	75.3 ± 0.800	1.96 ± 0.0140	2.50 ± 0.400	
TPS/PLA	30.2 ± 0.600	1.45 ± 0.0230	5.80 ± 1.90	41.5 ± 0.500	1.44 ± 0.0700	1.20 ± 0.200	
PP	39.4 ± 0.200	1.73 ± 0.0180	140 ± 110	52.0 ± 0.600	1.57 ± 0.0360	3.40 ± 0.100	
LDPE	21.6 ± 0.400	0.254 ±0.00800	40.0 ± 3.00	9.80	0.176 ± 0.00200	-	
PVC	48.2	3.31	21.0	-	-	545 (J/m)	[124]

**Table 6:** Mechanical properties of thermoset biopolymers and common fossil fuel derived thermosets. ^a^ Notched Izod.

Resin	Curing Agent	Tensile Strength (MPa)	Young’s Modulus (GPa)	Elongation at Break (%)	Flexural Strength (MPa)	Flexural Modulus (GPa)	Impact Strength (kJ/m^2^)	Ref.
ESO	TPAn	22.0	0.755	5.00	-	-	-	[104]
EP/ELO	PKA	20.3 ± 1.00	0.709 ± 0.00600	13.5 ± 2.00	-	-	4.99 ^a^ ± 0.100	[106]
EP/ECO	TETA	30.0 ± 3.00	1.24 ± 0.0200	32.0 ± 0.00	77.0 ± 4.00	2.12 ± 0.0200	21.2 ^a^ ± 2.00	[109]
BOF	44DDS	75.5	-	-	91.8	-	-	[125]
DGEI	DETA	52.0 ± 9.36	1.77 ± 0.142	5.00 ± 0.335	-	2.75	113 ^a^ ± 37.3	[113]
GEGA	IPDA	43.1	3.60	1.40	-	-	-	[120]
Epoxy	-	58.6	2.41	4.5	-	-	32.0 (J/m)	[124]
PUR	-	72.4	3.55	4.5	-	-	320 (J/m)	

**Table 7:** Thermal behaviour of injection moulded thermoplastic biopolymers and common fossil fuel derived polymers.

Name	T_g_ (°C)	T_m_ (°C)	Melt Flow Index (MFI)		Ref.
			g/10 min	oC/2.16 kg	
PLLA	71.4	163.4	-	-	[128]
PLA	51.0	145	3.69 ± 0.110	190	[121]
PGA	44.0	230	26.3 ± 1.23	228	
PCL	−62.0	70.0	16.6 ± 0.480	160	
PHB	5.00	180	-	-	[122]
CA	118	231	1.14 ± 0.0300	220	[121]
CAP	138	-	1.08 ± 0.0100	220	
TPS (PLA blended)	50.0	149	14.2 ± 1.42	170	[121]
PP	−4.00	166	-	-	[121]
LDPE	<−90	116	-	-	

**Table 8:** Thermal behaviour of thermoset biopolymers. ^a^ Carbon residue at 600 °C. ^b^ Carbon residue at 800 °C.

Resin	Curing Agent	T_g_ (oC)	T_d5_ (oC)	T_d10_ (oC)	T_d30_ (oC)	T_max_ (oC)	Carbon Residue (%)	Ref.
ESO	TPAn	48.4	-	-	-		-	[104]
EP/ELO	PKA	89.7	286	324	-	-	-	[106]
ECO	TETA	78.0	-	178	-	-	3.30 ^a^	[109]
BOF	44DDS	114	255	-	400	409	42.3 ^b^	[125]
DGEI	ISODA	50.0	228	269	328	-	5.20 ^a^	[113]
DGEVA	IPDA	97.0	-	-	-	361	19.0 ^a^	[118]
GEGA	IPDA	233	300	-	335	-	18.1 ^a^	[119]
Epoxy	Amine	136	332	363	-	-	-	[127]

**Table 9:** Mechanical properties of flax fibre bio-composites. UD—unidirectional, AM—aligned mat, RM0—random mat, CSM—chopped strand mat, WM—woven mat, QUD—quasi-unidirectional.

Composite	Fibre Arrangement	Fibre Content (%)	Young’s Mod (GPa)	Tensile Strength (MPa)	Elongation at Break (%)	Impact Strength (kJ/m^2^)	Ref.
Flax/PP	AM	29.0% vol	7.34 ± 0.330	143 ± 2.60	-	-	[146]
Flax/PLA	RM0	10.0% wt	3.90 ± 0.180	42.7 ± 1.29	-	9.97 ± 2.05	[147]
		20.0% wt	5.06 ± 0.0700	49.2 ± 1.40	-	10.5 ± 1.53	
		30.0% wt	6.31 ± 0.120	54.2 ± 4.57	-	11.1 ± 1.55	
Flax/PLA	RM0	30.0% wt	8.30 ± 0.600	53.0 ± 3.10	1.00 ± 0.200	-	[148]
		40.0% wt	7.30 ± 0.500	44.0 ± 7.20	0.900 ± 0.200	-	
Flax/PP		30.0% wt	5.00 ± 0.400	29.0 ± 4.20	2.70 ± 1.50	-	
		40.0% wt	7.60 ± 0.900	29.0 ± 3.10	1.50 ± 0.800	-	
Flax/PLA	UD	30.0% wt	10.8 ± 0.196	177 ± 14.0	1.70 ± 0.0840	-	[149]
Flax/PHB	CSM	30.0% wt	6.50	30.0	1.50	95.2 (J/m)	[150]
Flax/TPS	UD	50% wt	7.5	131	5.80	-	[151]
Flax/CAB	RM0	34.8% vol	5	44.9	1.20	-	[152]
Flax/Bio-epoxy	UD	50.0% vol	28.7	360	2.10	-	[153]
Flax/Epoxy	WM (2 × 2)	55.0% vol	9.20	120	3.00	-	[154]
Flax/Epoxy	WM (1 × 1)	36.8% wt	5.45 ± 0.160	52.7 ± 3.42	-	-	[155]
Flax/Epoxy	QUD	59% vol	33.3 ± 0.500	339 ± 18.0	1.72 ± 0.0800	-	[156]

**Table 10:** Thermal properties of flax fibre bio-composites.

Details	Fibre Content (%)	T_g_ (°C)	T_m_ (°C)	Crystallinity (%)	Ref.
Flax/PLA	30.0	62.5	153	-	[157]
Flax/PCL	10.0	−60.8	58.6	42.0	[158]
	20.0	−59.5	59.5	41.0	
	30.0	−59.2	59.3	37.0	
Flax/PHB	50.0	25.0	173	52.0	[159]

**Table 11:** Thermal degradation behaviour of flax fibre bio-composites.

Details	Fibre Content (%)	T_d5_ (°C)	T_d10_ (°C)	T_d50_ (°C)	Carbon Residue @ 600 °C (%)	Ref.
Flax/TPS	50.0	147	229	325	1.00	[151]
	80.0	95.7	251	354	4.30	
Flax/Epoxy	25.0	142	228	359	1.54	[160]
	35.0	155	253	351	1.12	
	45.0	131	200	351	0.720	
Flax/ELO	5.00	265	282	328	-	[161]
	20.0	260	282	333	-	

**Table 12:** Interfacial properties of flax fibre bio-composites.

Composite	Test Method	Embedded/Fragment Length (µm)	Apparent IFSS (MPa)	Ref.
Flax/PP	Pull-out	150	10.6	[163]
Flax/MA-PP	Pull-out	150	11.4	
Flax/Epoxy	Fragmentation	299	33.0 ± 7.00	[164]
Flax/MA-Epoxy	Fragmentation	364	24.0 ± 3.00	
Flax/Polyester	Fragmentation	703	13.0 ± 2.00	
Flax/Epoxy	Micro-bond	-	14.2 ± 0.400	[165]
Flax/Polyester	Micro-bond	-	22.7	

**Table 13:** Optimal NaOH treatment parameters for flax/fibre composites. PP—polypropylene.

Composite	Concentration (%)	Duration (Mins)	Change in Av. Longitudinal Properties (%)				Ref.
			Tensile Mod	Tensile Strength	Flex Mod	Flex Strength	
Flax/Epoxy	3.00	20.0	-	-	~+25.0	~+30.0	[175]
Flax/PP	5.00	120	+47.3	+22.8	+113	+146	[177]
Flax/Epoxy	5.00	30.0	+13.3	+21.9	+7.20	+16.1	[154]
Flax/PHBV	2.00	60.0	+10.5	+1.23	-	-	[178]
Flax/PFA	2.00	60.0	-	-	~+12.0	~+10.0	[179]
